# Evodiamine Induces Apoptosis and Inhibits Migration of HCT-116 Human Colorectal Cancer Cells

**DOI:** 10.3390/ijms161126031

**Published:** 2015-11-16

**Authors:** Lv-Cui Zhao, Jing Li, Ke Liao, Nian Luo, Qing-Qiang Shi, Zi-Qiang Feng, Di-Long Chen

**Affiliations:** 1 Laboratory of Stem Cell and Tissue Engineering, Department of Histology and Embryology, Chongqing Medical University, Chongqing 400016, China; 467202699zlc@gmail.com (L.-C.Z.); Lijingjj@gmail.com (J.L.); 467202699ln@gmail.com (N.L.); 467202699sqq@gmail.com (Q.-Q.S.); 467202699fzq@gmail.com (Z.-Q.F.); 2 Drug Engineering Research Center of Chongqing Medical University, Chongqing 400016, China; 3 Department of Respiration, Cheng Du Tumor Hospital, Chengdu 610041, China; E-Mail: 467202699lk@gmail.com

**Keywords:** Evodiamine (EVO), anti-cancer effects, molecular mechanisms, HCT-116 cells

## Abstract

Evodiamine (EVO) exhibits strong anti-cancer effects. However, the effect of EVO on the human colorectal cancer cell line HCT-116 has not been explored in detail, and its underlying molecular mechanisms remain unknown. In the present study, cell viability was assessed by Cell Counting Kit-8 (CCK-8). Cell cycle and apoptosis were measured by flow cytometry, and morphological changes in the nucleus were examined by fluorescence microscopy and Hoechst staining. Cell motility was detected by Transwell assay. ELISA was used to assess the protein levels of autocrine motility factor (AMF) in the cell supernatant, and protein expression was determined by Western blotting. Our results showed that EVO inhibited the proliferation of HCT-116 cells, caused accumulation of cells in S and G2/M phases, and reduced the levels of the secreted form of AMF. The protein levels of tumor suppressor protein (p53), Bcl-2 Associated X protein (Bax), B cell CLL/lymphoma-2 (Bcl-2), phosphoglucose isomerase (PGI), phosphorylated signal transducers and activators of transcription 3 (p-STAT3) and matrix metalloproteinase 3 (MMP3) were altered in cells treated with EVO. Taken together, our results suggest that EVO modulates the activity of the p53 signaling pathway to induce apoptosis and downregulate MMP3 expression by inactivating the JAK2/STAT3 pathway through the downregulation of PGI to inhibit migration of HCT-116 human colorectal cancer cells.

## 1. Introduction

Colorectal cancer is a common malignancy of the digestive tract, and its mortality rate is more than 30% [1]. Cancer metastasis, which is a characteristic of malignant tumors, is a leading cause of mortality in cancer patients [2]. Currently, chemotherapy is one of the main strategies for the treatment of colon cancer. However, chemotherapy is associated with side effects such as hair loss, bone marrow suppression, and drug-resistance, which decrease the benefit of chemotherapy for patients. The identification of efficient anticancer drugs with low toxicity has become an important objective of research. The HCT-116 cell line with high invasion in colon cancer cell lines, and the effect of Evodiamine (EVO) on HCT-116 cells are rarely reported. EVO, an alkaloid isolated from *Evodia rutaecarpa* Bentham (Rutaceae), has shown antitumor activity in a number of human cancers [3,4,5]. EVO possesses antitumor activities via inhibition of cell migration and invasion [6]. However, the metastasis inhibitory activity of EVO against human colorectal cancer cells and the underlying molecular mechanisms remain to be determined.

It is well known that tumor suppressor protein (p53) upregulated modulator of apoptosis (PUMA) is regulated by the tumor suppressor p53 [7]. B cell CLL/lymphoma-2 (Bcl-2)-binding component 3 (BBC3), a kind of PUMA, is a powerful direct activator of Bcl-2 Associated X protein (Bax), which is considered a pro-apoptotic protein [8]. Phosphoglucose isomerase (PGI), an important enzyme of the glycolytic and gluconeogenic pathways, catalyzes the inter-conversion of glucose-6-phosphate (G-6-P) into fructose-6-phosphate (F-6-P) [9]. PGI has been identified as an autocrine motility factor (AMF), and as such, it regulates tumor cell motility when secreted outside the tumor cell. Yasufumi *et al.* [10] reported that the silencing of AMF/PGI reduced cell growth, motility, invasion, and pulmonary metastasis.

The Janus kinase (JAK) signal transducer and activator of transcription 3 (STAT3) signal transduction pathway is activated by the binding of interleukin-6 (IL-6) to the IL-6 receptor (IL-6R) α and the recruitment of gp130, leading to the formation of a hexameric signaling complex. The JAK/STAT3 pathway plays important roles in cell proliferation, differentiation, survival, apoptosis, angiogenesis, and tumorigenesis [11,12,13].

Matrix metalloproteinases (MMPs) are a large family of zinc-containing endopeptidases that play important roles in several pathological processes including cancer cell metastasis. Wen *et al.* proposed that among MMP family members, the transcription, translation, and secretion of MMP3 are induced by AMF/PGI [14]. However, the mechanisms that lead to the induction of MMP3 expression are not fully understood. In addition, phosphorylated STAT3 directly binds to the MMP3 promoter region and regulates MMP3 expression [15]. Gao *et al.* provided evidence of the association between STAT3 and MMP3 in rheumatoid arthritis [16]. Both PGI and STAT3 are related to MMP3; however, the effect of PGI on the STAT3/MMP3 signaling pathway in HCT-116 cells remains unknown.

In the present study, we assessed the role of the p53 pathway, PGI, and the STAT3/MMP3 pathway in the anticancer effects of EVO in HCT-116 cells, and discussed the relationship between PGI and the STAT3/MMP3 pathway. Moreover, we firstly reported that PGI acts as an upstream signaling molecule of the STAT3/MMP3 pathway.

## 2. Results

### 2.1. Evodiamine (EVO) Suppresses Cell Proliferation and Causes Cell Cycle Arrest in HCT-116 Cells

**Figure 1:**
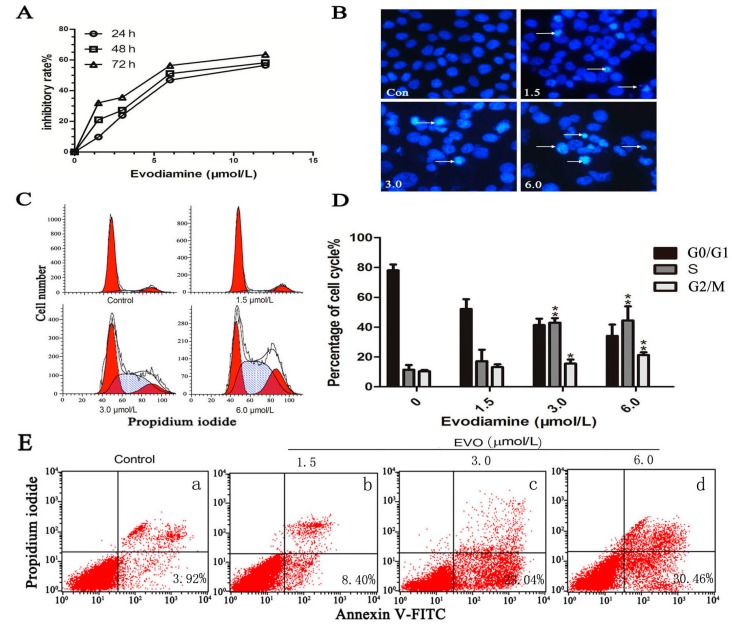
EVO shows anticancer effects in HCT-116 cells. (**A**) Cells were exposed to EVO at the indicated doses for 24, 48, and 72 h, and cell viability was assessed by the CCK-8 assay; (**B**) The nuclear morphological changes of apoptotic cells were observed after Hoechst staining. Arrows show pathological changes of apoptosis (original magnification, 400×); (**C**,**E**) HCT-116 cells were treated with various concentrations of EVO for 48 h, cell cycle arrest and apoptosis rate were analyzed by flow cytometry; and (**D**) * *p* < 0.05, ** *p* < 0.01 compared to control (0 μmol/L of EVO). Note: **a**: control, **b**–**d**: 1.5, 3.0, 6.0 μmol/L EVO, respectively (**E**).

The effect of EVO on HCT-116 cells was examined by assessing the proliferation of EVO-treated HCT-116 cells. EVO significantly reduced cell viability in a dose- and time-dependent manner (Figure 1A). Compared with the control group, EVO treatment for 48 h induced the typical nuclear morphological changes of apoptotic cells (Figure 1B). Apoptosis rate analysis showed that after the cells’ exposure to various concentrations of EVO for 48 h, the percentages of early apoptosis were gradually increased (Figure 1E). At high doses, EVO caused a significant accumulation of cells in the S (DNA synthesis phase) and G2/M (DNA postsynthetic phase and cell division phase) of the cell cycle (Figure 1C,D). With the exception of G0/G1 (stationary phase and the early stage of DNA synthesis phase).

### 2.2. Effect of EVO on Cell Cycle Regulatory Protein (Cyclin A1), and p53/Bax/Bcl-2 in HCT-116 Cells

**Figure 2:**
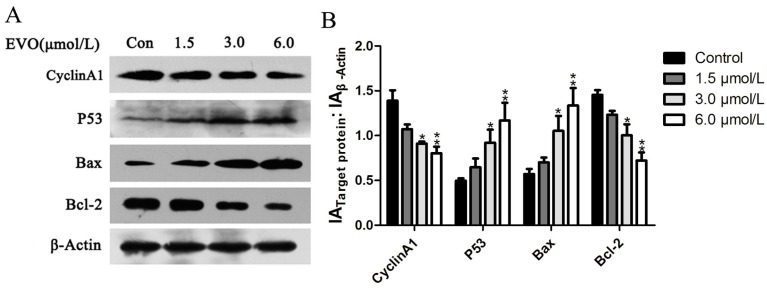
The effects of EVO on Cyclin A1 and the p53 signaling pathway. (**A**) HCT-116 cells were treated with the EVO for 48 h, and Cyclin A1 and p53 signaling pathway proteins were detected by Western blotting; (**B**) The relative expression ratio was calculated with Gray-scan value. The data shown represent mean ± SD (* *p* < 0.05, ** *p* < 0.01).

Next, we investigated the potential role of cell cycle arrest in the regulation of cell aacycle checkpoint proteins. For this purpose, the expression of the cell cycle regulatory protein (Cyclin A1) was examined. In addition, molecules in the p53 signaling pathway were also detected. As shown in Figure 2, Cyclin A1 and Bcl-2 were significantly downregulated, whereas p53 and Bax were upregulated in HCT-116 cells treated with EVO.

### 2.3. EVO Suppresses Expression of Phosphoglucose Isomerase (PGI), p-STAT3, MMP3, and PGI-Induced Migration in HCT-116 Cells

**Figure 3:**
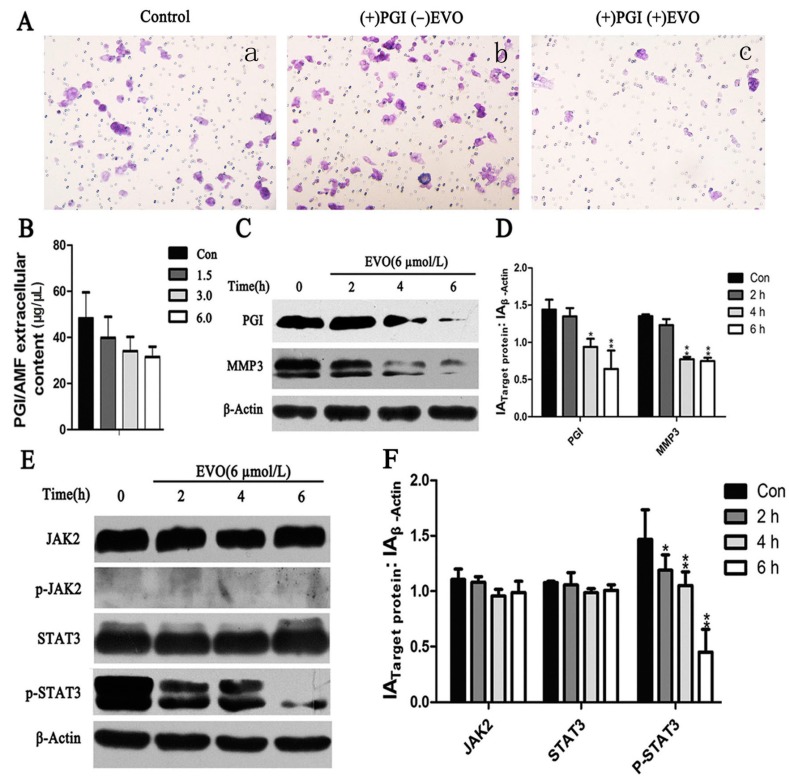
Migration ability, expression of PGI/AMF, JAK2/STAT3, and MMP3 were detected in HCT-116 cells treated with EVO. (**A**) Migration potential was assessed by Transwell assay after cells were treated by agents. Cells were seeded in Transwell insert upper part incubated with 10 μg/mL PGI/AMF (**b**) and with 10 μg/mL PGI/AMF + 6 μmol/L EVO (**c**) for 28 h, normal cells as the control group (**a**) (original magnification, 200×); (**B**) AMF was detected by ELISA after cells were treated with various concentrations of EVO for 6 h; (**C**–**F**) Cells were incubated with 6 μmol/L EVO for 2, 4, and 6 h, and the expression of PGI, MMP3 and JAK2/STAT3 was assessed. Relative expression ratio was calculated with Gray-scan value for (**D**,**F**). Data represent the results of three independent experiments (mean ± SD). Statistically different compared with the control (0 μmol/L of EVO) (* *p* < 0.05; ** *p* < 0.01).

AMF was first described as a cell motility-stimulating factor associated with cancer development and progression [17]. As shown in Figure 3A, EVO significantly inhibited PGI-induced migration in HCT-116 cells. The underlying mechanism was investigated by measuring the levels of extracellular AMF by ELISA. The results showed that AMF was downregulated by EVO in a dose-dependent manner (Figure 3B). Western blot analysis of PGI and MMP3 expression showed that EVO significantly downregulated PGI and MMP3 in a time-dependent manner (Figure 3C).

Whether EVO could inhibit STAT3 (Tyr705) activation in HCT-116 cells was examined. Cells were treated with EVO for the indicated times, and JAK2/STAT3 activation was detected by Western blotting. As shown in Figure 3E, EVO significantly inhibited STAT3 phosphorylation. These findings indicated that PGI, MMP3, and the JAK2/STAT3 signaling pathway may play a role in EVO-induced metastasis inhibition in HCT-116 cells.

### 2.4. Effect of the JAK2-Specific Inhibitor AG490 on JAK2/STAT3 Signaling, PGI and MMP3

**Figure 4:**
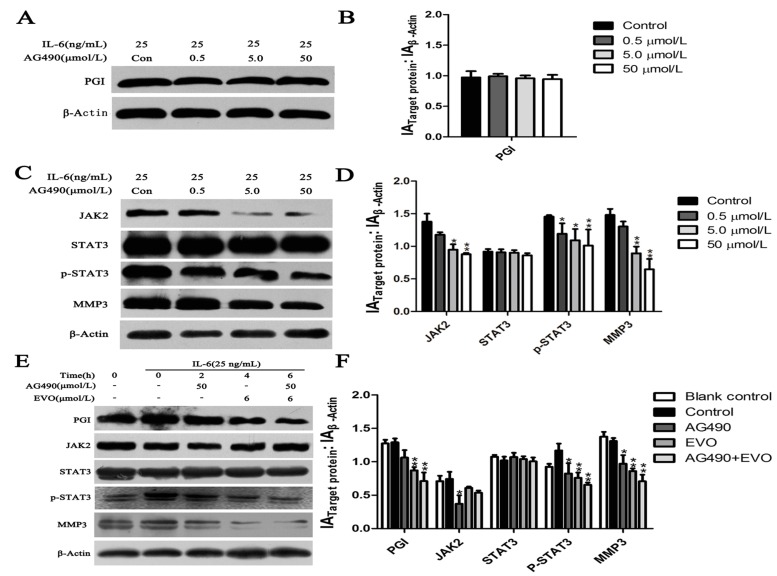
Effects of AG490 and EVO on PGI, the JAK2/STAT3 signaling pathway, and MMP3 in HCT-116 cells. (**A**,**C**) HCT-116 cells were treated with various concentrations of AG490 for 48 h and then stimulated with 25 ng/mL IL-6 for 15 min, after which whole-cell extracts of PGI, MMP3, JAK2, p-JAK2 (data not shown), STAT3, and p-STAT3 were analyzed by Western blotting; (**E**) HCT-116 cells were treated with 50 μmol/L AG490, 6 μmol/L EVO, and combined 50 μmol/L AG490 with 6 μmol/L EVO for 6 h, then stimulated with 25 ng/mL IL-6 for 15 min. Protein expression was analyzed by Western blotting; (**B**,**D**,**F**) The graph represents densitometry of the results of three independent experiments (mean ± SD). * *p* < 0.05, ** *p* < 0.01 compared to the control. The control group of Figure 4A–C with 25 ng/mL IL-6 + 0 μmol/L AG490, while Figure 4E with 25 ng/mL IL-6 + 0 μmol/L AG490 + 0 μmol/L EVO.

PGI activates the MMP3 promoter and phosphorylates STAT3 bound to the MMP3 protein [15]. Therefore, we assumed that there is a relationship between PGI, JAK2/STAT3, and MMP3. Because IL-6 is an activator of the JAK2/STAT3 signaling pathway, exogenous IL-6 was used to induce the phosphorylation of STAT3 at Tyr705. The relationship between PGI, JAK2-STAT3, and MMP3 was further examined by using AG490, a known JAK2/STAT3 inhibitor, to suppress IL-6-induced STAT3 (Tyr705) activation in HCT-116 cells. Cells were treated with different concentrations AG490 for 48 h, and then stimulated with 25 ng/mL IL-6 for 15 min. Western blot analysis showed that JAK2, p-STAT3, and MMP3 were significantly downregulated following treatment with AG490 (Figure 4C), whereas AG490 had no effect on the expression of PGI (Figure 4A).

### 2.5. The Inhibitory Effects of EVO on PGI, JAK2/STAT3 Signaling, and MMP3 Were Stronger than that of AG490

To compare the inhibitory effects of EVO and AG490 on STAT3 (Tyr705) activation, HCT-116 cells were treated with AG490 (50 μmol/L), EVO (6 μmol/L), and the combination of AG490 with EVO for different times. The results showed that EVO significantly inhibited the protein expression of PGI and IL-6-induced STAT3 (Tyr705) activation and MMP3, whereas AG490 exerted a marginal inhibitory effect. The combination of AG490 with EVO further inhibited the expression of PGI, p-STAT3, and MMP3 (Figure 4E).

### 2.6. EVO Abolishes PGI-Mediated STAT3/MMP3 Signal Transduction

**Figure 5:**
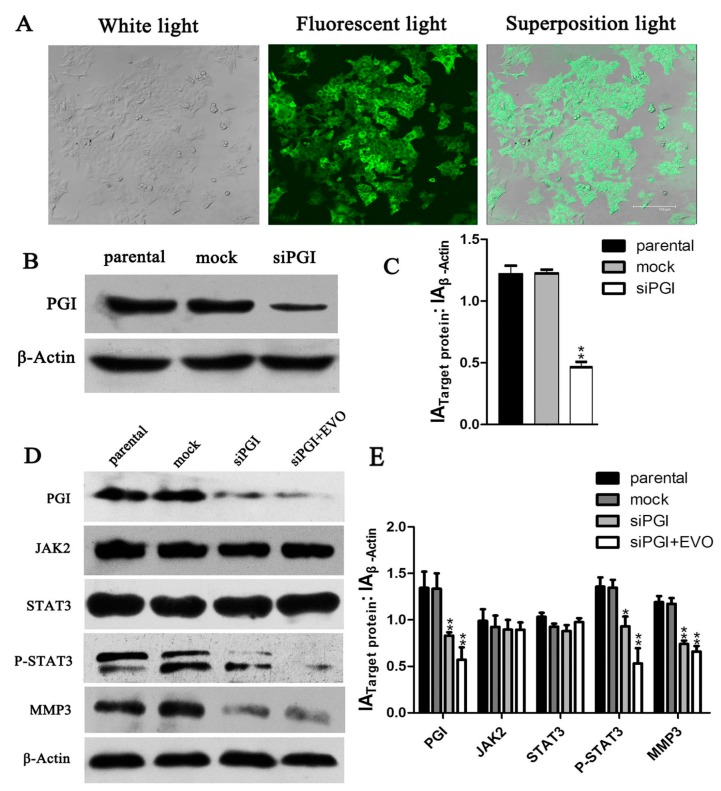
Expression of PGI, MMP3, and p-STAT3 in siPGI (HCT-116) cells. (**A**) Stably transfected siPGI (HCT-116) cells were screened (original magnification, 800×); (**B**) PGI expression in the stable cell line siPGI (HCT-116) was analyzed by Western blotting; (**D**) siPGI (HCT-116) cells were treated with 6 μmol/L EVO for 6 h, and proteins were subjected to Western blot analysis; (**C**,**E**), The graph represents the densitometry of the results of three independent experiments (mean ± SD), * *p* < 0.05, ** *p* < 0.01, *versus* the parental group.

To further validate the role of PGI in the inhibitory effect of EVO on migration and the relation between PGI and JAK2/STAT3/MMP3 in HCT-116 cells, we generated PGI-silenced HCT-116 cells siPGI (HCT-116). As shown in Figure 5A, laser scanning confocal microscopy showed that cells emitted green fluorescence, indicating successful lentivirus infection of HCT-116 cells. Western blot analysis showed that siPGI caused a 55% reduction of PGI expression (Figure 5C). Detection of protein levels of MMP3 and p-STAT3 in siPGI (HCT-116) cells showed a marked decrease in expression and p-STAT3 was further reduced by EVO (Figure 5D). These data demonstrate that PGI plays a pivotal role in the EVO-mediated inhibition of migration in HCT-116 cells. Data in Figure 4A–D together with those in Figure 5D suggested that PGI may act upstream of JAK2/STAT3, and MMP3 may act downstream.

## 3. Discussion

In the present study, we investigated the potential anticancer effects of EVO and elucidated the underlying mechanism in HCT-116 cells. PGI, MMP3, and p-STAT3 were downregulated in EVO-treated HCT-116 and siPGI (HCT-116) cells. In addition, the inhibitory effects of EVO on the JAK2/STAT3 signaling pathway and MMP3 expression were stronger than those of the JAK2-specific inhibitor AG490. EVO also showed an inhibitory effect on PGI, which was not observed for AG490. These findings suggested the involvement of other mechanisms mediating the suppression of IL-6-induced STAT3 (Tyr705) activation by EVO.

Studies have shown that EVO inhibits cell proliferation, induces apoptosis, and suppresses invasion and metastasis in a wide variety of tumor cells, including prostate cancer [18], colon cancer [19], leukemic T-lymphocyte [20], breast cancer [21], and hepatocellular carcinoma [22]. Shaw and his colleagues had reported that Cyclin A1 participated in the p53 signaling pathway, which is involved in apoptosis [23]. The results of the present study were consistent with previous findings, as some of the anticancer effects of EVO, such as reducing cell viability, inducing apoptosis, and arresting cells in the S and G2/M phases, were observed in HCT-116 cells (Figure 1). Bax and Bcl-2 are intrinsic signal molecules associated with the p53 pathway [8]. We found that the protein levels of p53 and Bax upregulated while Cyclin A1 and Bcl-2 activity decreased due to EVO in a concentration-dependent manner (Figure 2).

Metastasis is one of the characteristics of malignant tumors and determines the prognosis and survival of patients. PGI, a cytosolic enzyme of sugar metabolism that functions in the regulation of both glycolysis and gluconeogenesis, is closely related to tumor metastasis [24]. PGI is widely expressed in tumor tissues, and plays an essential role in enhancing tumor invasion and metastasis [25]. However, the effect of EVO on PGI has not been reported. In the present study, we provided evidence that EVO could abolish the PGI-mediated migration in HCT-116 cells (Figure 3A). In addition, EVO significantly inhibited the expression of PGI/AMF (Figure 3B,C). ELISA results showed no significant differences; however, this does not mean a lack of biological significance. It is possible that the amounts of extracellular AMF were low in HCT-116 cells. Yu *et al.* demonstrated that MMP3 acts as a downstream mediator in PGI/AMF-stimulated tumor cell metastasis [9]. Shih *et al.* reported that PGI activates the promoter of the MMP3 gene and induces cell migration [14]. In the present study, PGI silencing significantly inhibited the expression of MMP3 (Figure 5D). Further investigation into the mechanisms mediating the effect of PGI on MMP3 protein levels revealed the involvement of the JAK2/STAT3 signaling pathway.

STAT3 is associated with human malignancies [26], oncogenic transformation [27], angiogenesis [28], and invasion by tumor metastases [29]. The earliest time-point of STAT3 phosphorylation is 6 h, whereas 24 h is the peak time point at which STAT3 phosphorylation occurs [15]. In addition, the increase in PGI/AMF enzymatic activity follows a similar pattern as that of the phosphorylation level of STAT3 Tyr705, and the anti-apoptosis function of AMF/PGI can be completely abrogated by blocking STAT3 [30]. This indicates that PGI, STAT3, and MMP3 may be involved in the same signaling pathway. Our data showed that the expression of p-STAT3 and MMP3 were significantly reduced in siPGI (HCT-116) cells (Figure 5D), and AG490, a known JAK2/STAT3 inhibitor, reduced the levels of p-STAT3 and MMP3 without affecting PGI (Figure 4A). These results suggest that PGI functions upstream of JAK2/STAT3 and that MMP3 is a downstream effector.

STAT3, a member of the JAK/STAT family of proteins, is the most promising new target for cancer therapy. In addition, interleukin-6 (IL-6) and its family members were recently identified as regulators of JAK-STAT signaling in cancer [31]. We found that both EVO and AG490 inhibited IL-6-induced STAT3 phosphorylation. The inhibitory effects of EVO on JAK2/STAT3 signaling and MMP3 expression were stronger than those of AG490 (Figure 4E), suggesting that EVO is a strong inhibitor of tumor cell migration in colon cancer. The anti-proliferative effect of EVO is associated in part with the induction of G2/M phase cell cycle arrest and apoptosis in human lung cancer cells [32,33]. Our study showed that EVO exhibited potent anti-proliferative activity and induced cell cycle arrest at the S and G2/M phases in HCT-116 cells (Figure 1D). The EVO-induced cell cycle arrest was consistent with the inhibition of the S and G2/M phase block exclusive protein Cyclin A1 (Figure 2A). Shaw *et al.* [23] reported that Cyclin A1 is involved in apoptosis and growth arrest downstream of p53. EVO significantly upregulated p53 and increased the Bax/Bcl-2 ratio, suggesting that EVO induced apoptosis via the intrinsic apoptotic pathway (Figure 2A). These findings suggest that the anti-proliferative effect of EVO is associated with the induction of S phase cell cycle arrest and apoptosis.

Taken together, the antitumor activity of EVO may be mediated by the inhibition of p53 signaling and the inactivation the STAT3/MMP3 signaling pathway mediated by the downregulation of PGI expression in the early stage of tumor development. These results provide important information supporting the use of an efficient natural medicine or prodrug for colorectal carcinoma therapy.

## 4. Experimental Section

### 4.1. Cell Culture

The human colorectal carcinoma cell line (HCT-116 cells) was a gift from the Gene Research Division of the Third Military Medical University Institute, Chongqing, China. Cells were cultured with Dulbecco’s Modified Eagle Medium (DMEM) and 10% fetal bovine serum at 37 °C in a 5% CO_2_ incubator.

### 4.2. PGI Silencing

HCT-116 cells were cultured in six-well plates for 24 h before transfection. Cells at 70% confluence were transfected with PGI silencing lentiviral vector (Neuron Biotechnology Company, Shanghai, China). The primer sequences were as follows: forward primer, 5′-GATCCGCGGTACCGCGAGCACCGCTTTCAAGAGAAGCGGTGCTCGCGGTACCATTTTTTGGAAA-3′; reverse primer, 5′-AGCTTTTC CAAAAAATGGTACCGCGAGCACCGCTTCTCTTGAAAGCGGTGCTCGCGGTACCACG-3′. Cells stably transfected with siPGI (HCT-116) were screened with puromycin. HCT-116 cells and siPGI (HCT-116) cells were maintained in DMEM (Gibco, Grand island, NY, USA) and cultured in a humidified atmosphere in a 5% CO_2_ incubator at 37 °C.

### 4.3. Reagents and Antibodies

Evodiamine (EVO) was purchased from Nanjing Zelang Pharmaceutical and Biological Products Company (Nanjing, China). A 100 μmol/L solution of EVO was prepared in dimethyl sulfoxide (DMSO) and stored at 4 °C. Cell Counting Kit-8 was purchased from Dojindo (Kumamoto, Japan). Hoechst 33258 was purchased from Beyotime Institute of Biotechnology (Shanghai, China). Human Glucose-6-phosphate Isomerase was purchased from Prospec (Ness Ziona, Israel). The 8.0 μm pore diameter Transwell motility chambers were purchased from Corning (Corning Costar, New York, NY, USA). The human GPI ELISA kit was purchased from Cusabio (Wuhan, China). Ultrafiltration centrifuge tubes were purchased from Millipore (Merck, Kenilworth, NJ, USA). AG490 was obtained from Tocris Bioscience (Bristol, UK). IL-6 was purchased from Peprotech (Rocky Hill, NJ, USA). STAT3, phospho-JAK2, and β-actin antibodies were purchased from Cell Signaling Technology. Phospho-STAT3, JAK2, MMP3, PGI antibodies were purchased from Abgent (San Diego, CA, USA). P53, Bcl-2, Bax, and Cyclin A1 antibodies were purchased from Abcam.

### 4.4. Cell Viability Analysis

Cell viability was measured by the Cell Counting Kit-8 (CCK-8). HCT-116 cells were seeded in a 96-well plate at a density of 1 × 10^4^ cells in 100 μL complete culture medium. After 24 h, cells were treated without or with EVO at various concentrations (ranging between 1.5 and 12 μmol/L, for 24, 48, and 72 h). CCK-8 (20 μL) was added and then incubated for 3 h at 37 °C. The optical density of each sample at 450 nm was measured using a microplate reader (Bio-Rad, Hercules, CA, USA). Cells treated with 0.1% DMSO served as a solvent control. Complete culture medium without cells served as a blank control. All experiments were performed in triplicate. The drug concentration resulting in 50% inhibition of growth (IC_50_) was determined.

### 4.5. Cell Cycle and Apoptosis Rate Analysis

HCT-116 cells were seeded at a density of 2 × 10^5^/mL in six-well plates. After 24 h, the cells were exposed to different concentrations (0, 1.5, 3.0, and 6.0 μmol/L) of EVO for 48 h. Cell cycle analysis: the treated cells were then fixed in 70% ethanol for 24 h at 4 °C, collected and centrifuged (1500× *g*). The cell pellet was resuspended in Phosphate buffered saline (PBS) (400 μL), RNaseA (10 mg/mL, 50 μL), and Propidium iodide (PI) (2 mg/mL, 10 μL). The mixtures were incubated in the dark at 37 °C for 30 min. Apoptosis rate analysis: the treated cells were collected and washed twice with PBS to remove the medium. At least 1 × 10^5^ cells were resuspended in 100 μL binding buffer containing Annexin V-FITC and propidium iodide (PI) according to the manufacturer’s protocol (Beyotime Institute of Biotechnology, Shanghai, China). Then the cell cycle and apoptosis rates were analyzed with FAC-Scan laser flow cytometry (FAC-S, Calibur, Becton Dickinson, Franklin Lakes, NJ, USA). Data were analyzed using CELL Quest software.

### 4.6. Hoechst Staining

Cells were plated on six-well chamber slides, and allowed to attach. After being exposed to different concentrations of EVO for 48 h, they were then washed three times with PBS, fixed with 4% paraformaldehyde for 10 min at 37 °C, and stained with 50 μL Hoechst 33258 staining solution for 15 min in dark. The cells were pictured under a fluorescence microscope (Olympus, Tokyo, Japan). Characteristic apoptotic morphology such as cell shrinkage and nuclear pale was observed after Hoechst staining, while the nuclear of non-apoptotic cells was stained a homogenous blue color.

### 4.7. Transwell Assay

Migration ability of cells was evaluated by Transwell assay. This assay examined the ability of the cells moved to the underside of the membrane filter. The EVO-treated cells and the normal cells were seeded in the top part of the chamber at a density of 6 × 10^4^ cells/(100 μL), respectively, and grown on 8.0 μm porous polycarbonate membranes. The bottom part of the chamber was filled with DMEM or with medium supplemented with the presence or absence of human glucose-6-phosphate isomerase (PGI). After 28 h, cells remaining on the upper surface of the filter were removed using cotton tips and the cells invading the lower side of the membrane were fixed with 4% paraformaldehyde for 30 min, followed by staining with crystal violet for 30 min. The cells in three random fields of view at a magnification of ×200 were observed.

### 4.8. Immunoblot Assay

Cells were cultured in six-well plates at a density of 5 × 10^5^ cells/mL in a 5% CO_2_ incubator at 37 °C for 24 h and treated with or without EVO. Total protein was prepared according to the manufacturer’s instructions of BCA Protein Assay Kit (Beyotime Biotechnology, Haimen, China). Equal amounts of proteins from each sample were separated by sodium dodecyl sulfate-polyacrylamide gel electrophoresis (SDS-PAGE), and separated proteins were transferred to polyvinylidene difluoride (PVDF) membranes. The membranes were blocked with 5% non-fat milk or 5% bovine serum albumin (BSA) for 2 h, incubated with primary antibody overnight at 4 °C, washed in Tris-buffered saline with Tween 20 (TBST) for 30 min, and incubated with IgG horseradish peroxidase (HRP)-conjugated secondary antibody for 1.5 h at room temperature. Bound immune-complexes were detected by enhanced chemiluminescence (ECL) Western blotting detection reagents and exposure to X-ray film. PGI, JAK2, STAT3, p-STAT3, and MMP3 expressions were normalized against that of β-actin.

### 4.9. Enzyme-Linked Immunosorbent Assay (ELISA)

Variations in AMF protein levels in the supernatant of cells treated with different concentrations of EVO for 6 h were quantified by ELISA. Briefly, equal amounts of culture supernatant were collected and the samples were concentrated in ultrafiltration centrifuge tubes (15,000 rpm at 4 °C for 20 min). Then, samples were allowed to react with anti-PGI/AMF on the surface of microplate wells. Plates were incubated, washed, and bound immune-complexes were treated with peroxidase-labeled Fab’ anti-PGI/AMF antibody; bound peroxidase-labeled antibody was quantified by addition of Tetramethylbenzidine (TMB) substrate, and Optional density (OD) values were detected at 450 nm in a microplate enzyme-linked immunosorbent assay detector.

### 4.10. Statistical Analysis

Statistical differences were analyzed using Student’s *t*-test with SPSS 22.0 software. *p* < 0.05 was considered statistically significant. Values are expressed as the mean ± SD. Three or more separate experiments were performed.
